# The Reliability of a Semi-automated Algorithm for Detection of Cortical Interruptions in Finger Joints on High Resolution CT Compared to MicroCT

**DOI:** 10.1007/s00223-017-0264-5

**Published:** 2017-03-28

**Authors:** M. Peters, A. Scharmga, A. van Tubergen, J. Arts, D. Loeffen, R. Weijers, B. van Rietbergen, P. Geusens, J. P. van den Bergh

**Affiliations:** 1 Division of Rheumatology, Department of Internal Medicine, Maastricht University Medical Center, P.O. Box 5800, 6202 AZ Maastricht, The Netherlands; 2 Research School CAPHRI, School for Public Health and Primary Care, Maastricht, The Netherlands; 3 NUTRIM School of Nutrition and Translational Research in Metabolism, Maastricht University, Maastricht, The Netherlands; 4 Department of Orthopaedic Surgery, Maastricht University Medical Center, Maastricht, The Netherlands; 5 Faculty of Biomedical Engineering, Eindhoven University of Technology, Eindhoven, The Netherlands; 6 Department of Radiology, Maastricht University Medical Center, Maastricht, The Netherlands; 7 Faculty of Medicine and Life Sciences, Hasselt University, Hasselt, Belgium; 8 Department of Internal Medicine, VieCuri Medical Center, Venlo, The Netherlands

**Keywords:** Cortical interruptions, High resolution peripheral quantitative computed tomography, Micro computed tomography, Finger joints, Rheumatoid arthritis

## Abstract

We developed a semi-automated algorithm for the detection of cortical interruptions in finger joints using high-resolution peripheral quantitative computed tomography (HR-pQCT). Here, we tested its reliability compared to microCT (µCT) as gold standard. Nineteen joints of 10 female anatomic index fingers were imaged by HR-pQCT and µCT (82 and 18 µm isotropic voxel sizes, respectively). The algorithm was applied for detection of cortical interruptions of different minimum diameters (range >0.16 to >0.50 mm). Reliability was tested at the joint level with intra-class correlation coefficient (ICC) for the number of interruptions and interruption surface, and at the level of a single interruption for matching between HR-pQCT and µCT with a fixed interruption diameter (>0.10 mm) on µCT. The positive predictive value (PPV_0.10mm_) and sensitivity_0.10mm_ were evaluated. The mean number of interruptions per joint depended on the diameter cut-off and ranged from 3.4 to 53.5 on HR-pQCT and from 1.8 to 45.1 on µCT for interruptions >0.50 to >0.16 mm, respectively. Reliability at the joint level was almost perfect (ICC ≥0.81) for both the number and surface of interruptions >0.16 and >0.33 mm. As expected, the PPV_0.10mm_ increased with increasing interruption diameter from 84.9 to 100%, for interruptions >0.16 and >0.50 mm, respectively. However, the sensitivity_0.10mm_ decreased with increasing interruption diameter from 62.4 to 4.7%. This semi-automated algorithm for HR-pQCT in finger joints performed best for the detection of cortical interruptions with a minimum diameter of >0.16 or >0.33 mm, showing almost perfect reliability at the joint level and interruptions matched with those on µCT.

## Introduction

Peri-articular cortical interruptions are one of the characteristic features of bone involvement in rheumatoid arthritis (RA) and predictors of further radiographic progression [1, 2]. Conventional radiographs (CR) are considered the gold standard for detection of cortical interruptions (i.e. erosions) in the hand joints in RA in daily practice. However, CRs have limited sensitivity to visualize joint damage compared to computed tomography (CT), MRI and ultrasound (US) [3–6].

High-resolution peripheral quantitative CT (HR-pQCT) is a non-invasive imaging method for in vivo three-dimensional (3D) characterization of human bone [7]. Studies with the HR-pQCT in finger joints have shown that it has a higher sensitivity for detection of cortical interruptions compared with US, CR, and MRI [8–11]. Studies that compare HR-pQCT imaging to microCT (µCT) imaging with a higher resolution in the detection of cortical interruptions in finger joints are, however, scarce [12].

The cortical bone in finger joints is thin and low mineralized. Cortical bone regions of approximately 100 µm can be observed [13]. The accuracy of detecting these thin cortices might be lower using HR-pQCT because of its spatial resolution of 130 µm [14]. In regions of thin cortical bone, i.e. in the range of the spatial resolution, the bone density of the cortex will be underestimated due to the partial volume effect. After thresholding the image, this region might appear as a non-bone region (i.e. a false cortical interruption) in the binary image.

Recently, we developed a semi-automated algorithm that reliably detects small cortical interruptions in finger joints on HR-pQCT images [15]. However, the algorithm uses binary images to detect cortical interruptions, and might therefore be susceptible for false detection of cortical interruptions. Increasing the minimum cut-off diameter of interruptions, might be a solution to avoid false detection of cortical interruptions.

Therefore, the aim of this study was to investigate the reliability of the algorithm on HR-pQCT for different minimum diameters of cortical interruptions to detect its presence and to quantify the surface of cortical interruptions compared to µCT, as gold standard.

## Materials and Methods

### Specimens

For this study, we used anatomic specimens, because μCT imaging can only be executed in-vitro. Metacarpophalangeal (MCP) and proximal interphalangeal (PIP) joints of ten female right hand human anatomic index fingers were imaged by both HR-pQCT and μCT. One PIP joint could not be evaluated due to a failed μCT scan, due to bad fixation. Thus, 10 MCP and 9 PIP joints remained for analysis. The anatomic specimens were obtained from the Department of Anatomy and Embryology, University of Amsterdam, Amsterdam, the Netherlands. A handwritten and signed codicil from each donor posed when still alive and well, is kept at the Department of Anatomy and Embryology, University of Amsterdam, Amsterdam, The Netherlands. The medical history of the donors was unknown. The fingers were fixated in formalin.

### HR-pQCT and µCT Image Acquisition

HR-pQCT (XtremeCT, Scanco Medical AG, Switzerland) scans were performed at clinical in vivo settings, i.e. at 60kVp tube voltage, 900 µA tube current, 100 ms integration time and 82 µm voxel size. For the MCP joint, a reference line was placed on top of the metacarpal head, covering an area of 12.00 mm in proximal direction and 6.04 mm in distal direction (total scan area 18.04 mm, 220 slices). For the PIP joint, a reference line was placed on top of the proximal phalanx, covering an area of 6.00 mm in proximal direction and 3.02 mm in distal direction (total scan area 9.02 mm, 110 slices).

µCT (µCT 80, Scanco Medical AG, Switzerland) scans were performed with settings: 70kVp tube voltage, 114 µA tube current, 300 ms integration time and 18 µm voxel size. For the MCP joint, a reference line was placed on top of the metacarpal head, covering an area of 10.00 mm in proximal direction and 5.26 mm in distal direction (total scan area 15.26 mm, 848 slices). For the PIP joint, a reference line was placed on top of the proximal phalanx, covering an area of 6 mm in proximal direction and 3.45 mm in distal direction (total scan area 9.45 mm, 525 slices).

Differences in the extent of the scanned areas as well as in joint angles were noticed because the fingers were scanned horizontally on HR-pQCT and vertically on μCT. Corresponding first and last slices of the overlapping region were visually determined to ensure that the same region of interest was used in the detection of cortical interruptions on both imaging modalities. In this overlapping region, the total, cortical and trabecular volumetric bone mineral density (Tot.BMD, Ct.BMD and Tb.BMD, respectively) of the specimens were calculated from the HR-pQCT scans to get insight into the bone mineralization of the specimens.

### Cortical Interruption Detection

#### The Algorithm

A semi-automated algorithm for the detection of cortical interruptions was applied on both the HR-pQCT and µCT images. The algorithm is described elsewhere [15]. In short, *first*, the outer margin of the cortex was identified according to a modified auto-contouring algorithm originally developed for periosteal segmentation of the distal radius and tibia [15–17]. The contour was visually corrected by an operator if necessary. *Second*, a binary segmentation into bone and non-bone was performed. For the HR-pQCT datasets, the bone was segmented using the standard evaluation protocol from the manufacturer for radius and tibia, which included Laplace-Hamming filtering and thresholding [18]. Segmentation of the µCT dataset was performed using a Gaussian filtering (sigma = 0.8, support = 1 voxel) with a constant threshold of 247 per 1000 of the maximum possible voxel value, to equal bone volume to total volume (BV/TV) of the µCT with that of the HR-pQCT. *Third*, a cortical mask with a constant thickness was generated to identify the cortical bone. This was 4 voxels (=0.328 mm) for HR-pQCT and 18 voxels (=0.324 mm) for µCT, because this approached the average cortical thickness in MCP joints images [15]. *Fourth*, the cortical bone was analyzed for discontinuities that meet the preset criteria of the minimum diameter of a cortical interruption (>0.16, >0.33 and >0.50 mm). The total number of detected cortical interruptions and interruption surface per joint were analyzed.

#### Diameter Selection

The minimum diameter of an interruption was selected by the number of dilation steps. This way, we ensured that the interruption had an opening through the cortex with this minimum diameter and at both the periosteal and endosteal boundary of the cortical mask.

**Fig. 1:**
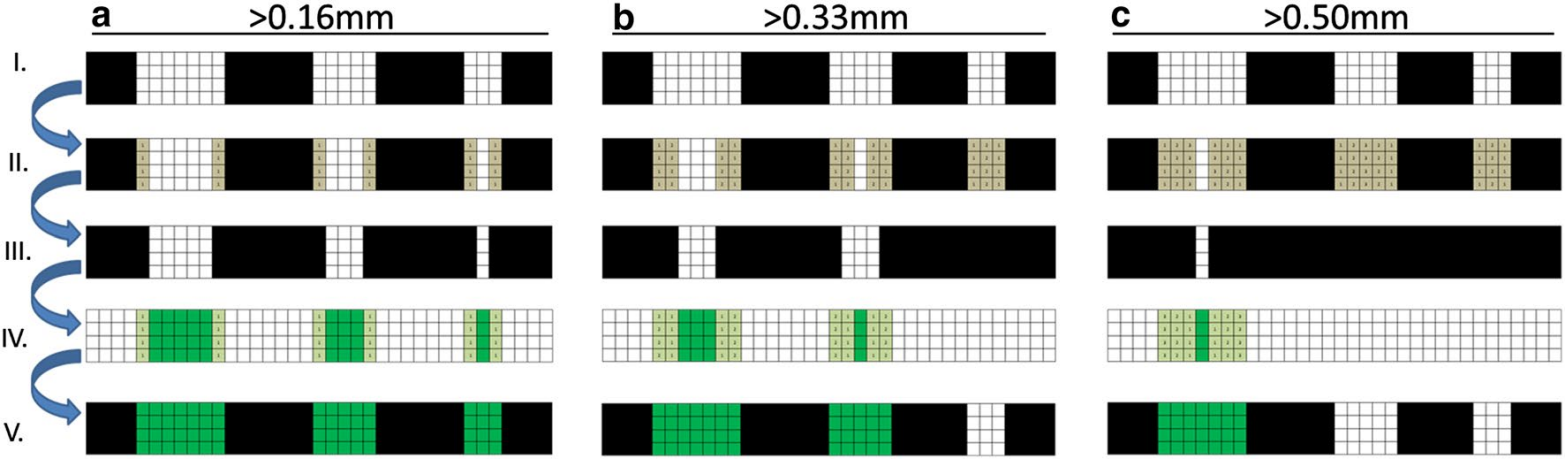
Two-dimensional examples of cortical interruptions with different diameters on HR-pQCT voxel level, and the multiple dilation steps that were used to obtain different minimum diameters of interruptions. The following steps are made by the algorithm: (I) shows the original cortex (*black*) and cortical interruptions (*white*). The cortex is then dilated with 1 (**a**), 2 (**b**) or 3 (**c**) voxels (II, *grey*). The dilated cortex (III) is then inverted, and interruptions connected to both the endosteal and periosteal boundary are selected. Finally, the selected interruptions are dilated to its original size (IV), and displayed in the original cortex (V). Shown is that with the increase in the number of dilation steps, only interruptions with a larger diameter are detected (V, *green*). (Color figure online)

Two-dimensional examples of cortical interruptions on HR-pQCT voxel level and the five steps performed by the algorithm are shown in Fig. 1 I–V. We used sequentially one, two, and three dilation steps resulting in minimum diameters of an interruption of >0.16 mm (Fig. 1a), >0.33 mm (Fig. 1b) and >0.50 mm (Fig. 1c), respectively. Shown is that with the increase in the number of dilation steps, only interruptions with a larger diameter are detected (Fig. 1 V, green).

For μCT the number of dilations was chosen such that it resulted in similar minimum diameters (Table 1). In addition, we were interested if an interruption that was detected with the algorithm on HR-pQCT was present on µCT not taking into account the minimum interruption diameter. Moreover, we were interested in how many interruptions would be missed when increasing the minimum diameter on HR-pQCT compared to the total number of interruptions >0.10 mm detected on µCT. Therefore, for the μCT only, we also set the minimum interruption diameter to >0.10 mm. All investigated dilation steps and corresponding minimum diameter values on HR-pQCT and µCT are shown in Table 1.

**Table 1:** Parameters of the algorithm for the different minimum diameters of the interruptions on HR-pQCT and µCT

Minimum diameter of the interruption	Number of dilation steps in HR-pQCT	Diameter of the interruptions	Number of dilation steps in µCT	Diameter of the interruptions
>0.10 mm	–	–	3 (0.054 mm)	>6 (0.108 mm)
>0.16 mm	1 (0.082 mm)	>2 (0.164 mm)	5 (0.090 mm)	>10 (0.180 mm)
>0.33 mm	2 (0.164 mm)	>4 (0.328 mm)	9 (0.180 mm)	>18 (0.324 mm)
>0.50 mm	3 (0.246 mm)	>6 (0.492 mm)	14 (0.252 mm)	>28 (0.504 mm)

Minimum diameters of the interruptions are ranging from 0.10 to 0.50mm and their corresponding number of dilated voxels on the HR-pQCT and µCT are shown. Values are displayed as number of voxels (mm)

#### Registration of HR-pQCT and µCT Images

**Fig. 2:**
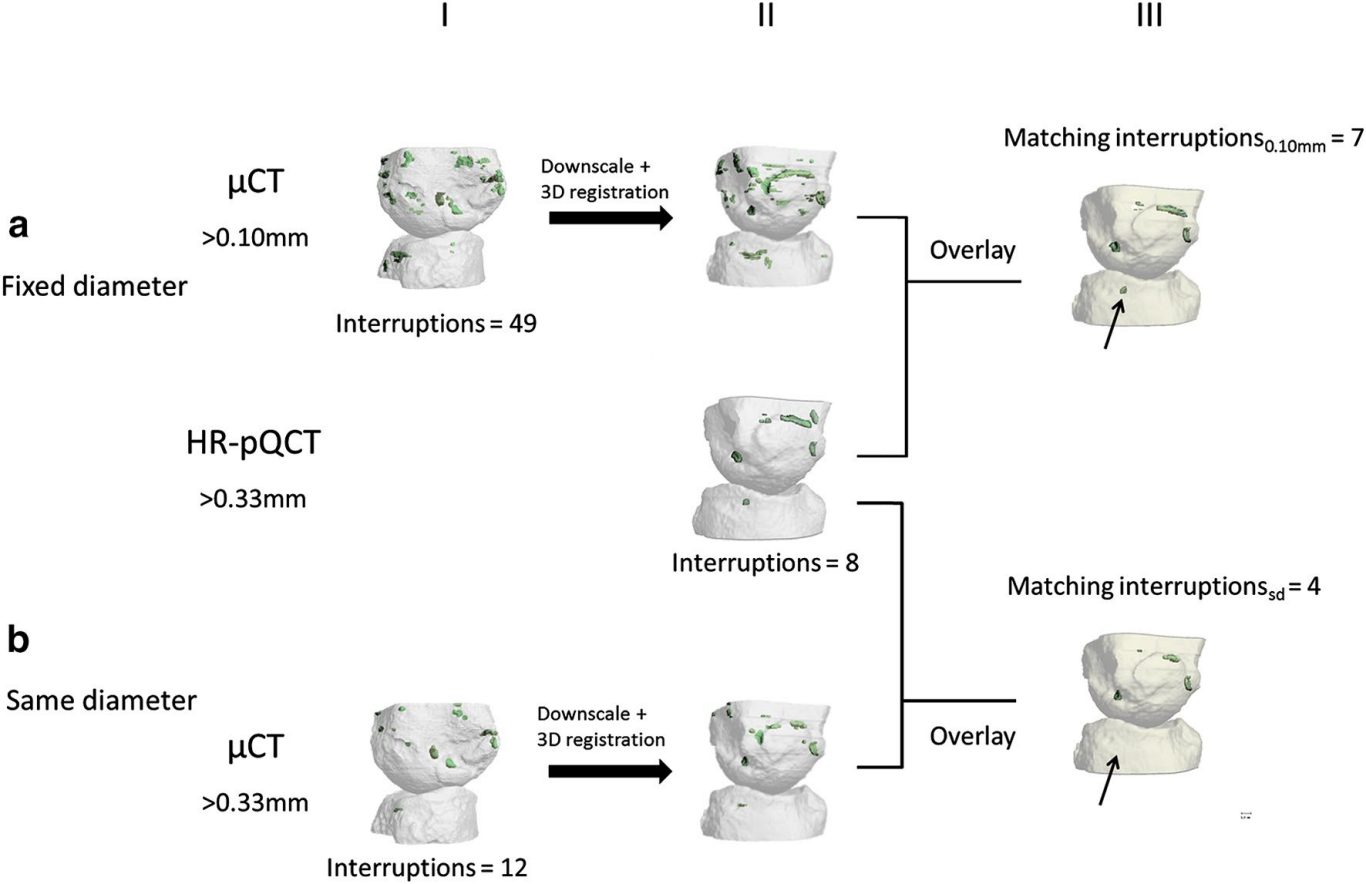
Matching interruptions between HR-pQCT and µCT with a variable minimum diameter on HR-pQCT and fixed minimum diameter of >0.10 mm on µCT (**a**), and with the same variable minimum diameter on HR-pQCT and µCT (**b**). *I* Represents the outputs of the cortical interruption algorithm on µCT. The µCT image is downscaled and registered to the HR-pQCT output image (*II*). The µCT and HR-pQCT images are overlayed, and interruptions with at least 20 HR-pQCT voxels overlap are shown in (*III*). The *black arrow* indicates an interruption that was found matching on HR-pQCT and µCT in (**a**) with a fixed minimum diameter on µCT (matching interruptions_0.10mm_), but was not found matching with the same minimum diameter (**b**, matching interruptions_sd_)

To evaluate matching interruptions on exactly the same location on HR-pQCT and µCT, a 3D rigid image registration was performed (Fig. 2). Each bone of the joint was registered separately to avoid registration errors due to different joint angles. The µCT images of the interruptions detected were downscaled and transformed to the HR-pQCT images (Fig. 2 II). The μCT and HR-pQCT images are overlayed, and only interruptions that overlapped with at least 20 voxels (=0.011mm^3^) were counted as matching (Fig. 2 III). This is the smallest interruption volume that can be detected on HR-pQCT images for interruptions >0.16 mm (15).

The number of matching interruptions was calculated for the minimum diameters of >0.16, >0.33 and >0.50 mm on HR-pQCT with a fixed minimum diameter of the interruption of >0.10 mm on µCT (matching interruption_0.10mm_) (Fig. 2a). In addition, the number of matching interruptions was calculated for minimum diameters of >0.16, >0.33 and >0.50 mm on HR-pQCT with the same minimum diameter on µCT (matching interruptions_sd_) (Fig. 2b).

### Statistical Analysis

Descriptive statistics were calculated for the number of interruptions and interruption surface per joint. Differences between the imaging modalities were analyzed with Wilcoxon signed-rank test. On the joint level, the reliability of the algorithm for different minimum diameters of the interruptions was estimated by ICC with a two-way random model and absolute agreement.

On the single interruption level, reliability of the HR-pQCT with different minimum diameters was evaluated with positive predictive values (PPV, Eq. 1) with a fixed µCT diameter of >0.10 mm (PPV_0.10mm_) and same minimum diameter (PPV_sd_) of interruptions. In addition, reliability of single interruptions on the HR-pQCT with different minimum diameters were evaluated with the sensitivity (Eq. 2) for detection of interruptions >0.10 mm on µCT (sensitivity_0.10mm_), and the sensitivity for detection of interruptions with the same minimum diameter (sensitivity_sd_). Statistical analysis was performed using IBM SPSS Statistics for Windows, Version 20.0 (IBM Corp., Armonk, NY).

1 $$PP{V_{0.10\text{mm}{\,} \text{or}{\,} \text{sd}}} = \frac{{ \text{Matching}\, \text{interruptions}_{0.10\text{mm}\, \text{or}\, \text{sd}}}}{{\text{Nr.}\, \text{of}\, \text{interruptions}_{HRpQCT}}} \times 100\%$$

2 $$\text{Sensitivity}_{0.10\text{mm}\, \text{or}\, \text{sd}} = \frac{{ \text{Matching}\, \text{interruptions}_{0.10\text{mm}\, \text{or}\, \text{sd}}}}{{\text{Nr.}\, \text{of}\, \text{interruptions}_{\mu CT}}} \times 100\%$$

## Results

The mean age (SD) of the donors was 85.1 (9.6) years, the medical history was unknown. The average Tot.BMD was 248.5mgHA/cm^3^ for the joints. The Ct.BMD and Tb.BMD were 464.0 and 179.7 mgHA/cm^3^, respectively.

### Visual Evaluation of HR-pQCT and µCT Examinations

**Fig. 3:**
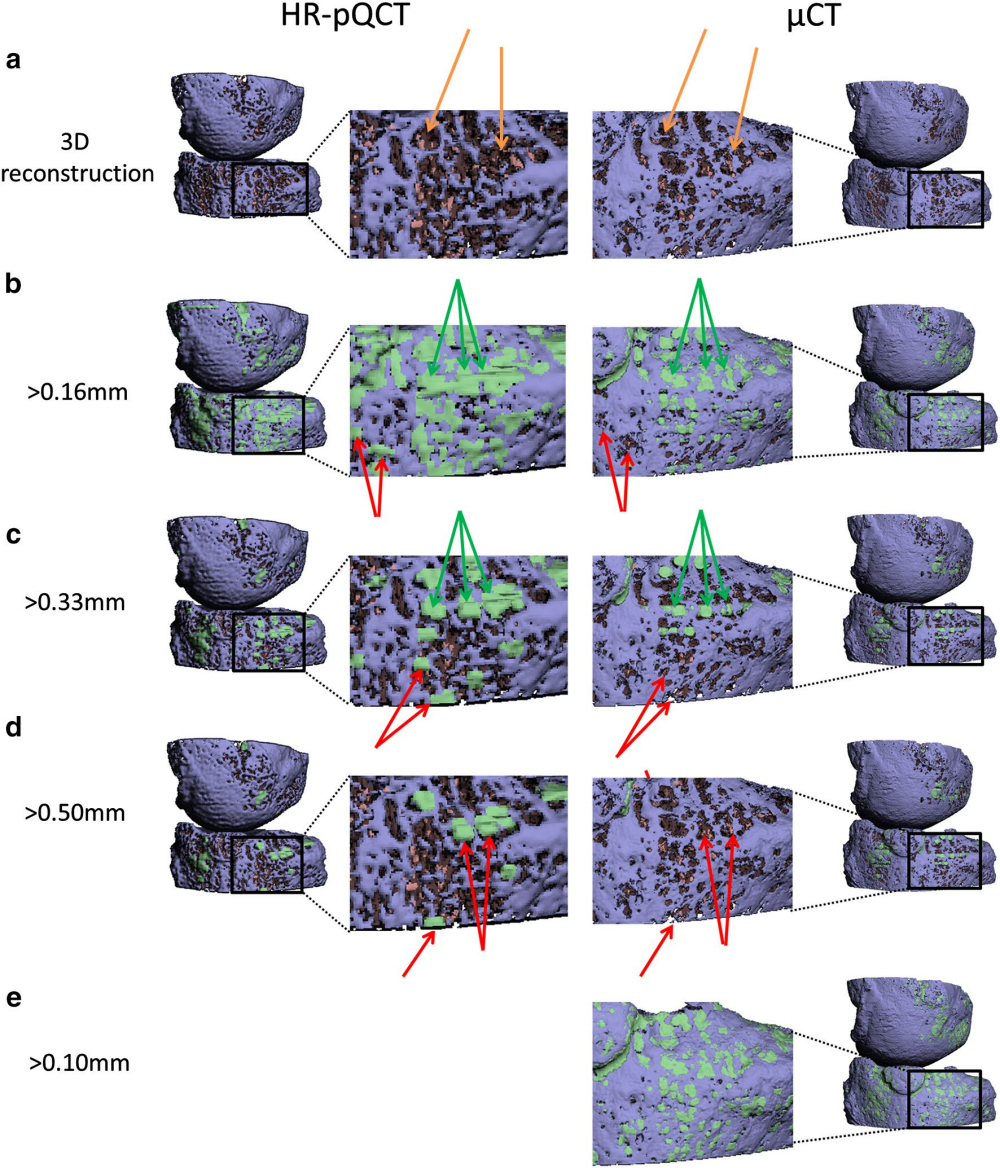
Typical example of 3D reconstructions of an MCP joint of a HR-pQCT scan (*left*) and µCT scan (*right*) and the 3D outputs of the algorithm for the different minimum diameters of the interruptions. The cortical region is indicated in purple, trabecular region in *orange*, and the cortical interruptions that were detected by the algorithm are shown in green. **a** Shows that the cortex is very porous on the HR-pQCT and µCT scan, and that thin cortices were identified on µCT, but not on HR-pQCT (*orange arrows*). **b** Shows that most pores were identified as cortical interruptions on HR-pQCT and µCT. In addition, most cortical interruptions detected on HR-pQCT were also detected on µCT (*green arrows*), with some exceptions (*red arrows*). **c** Shows that only larger pores are identified as cortical interruptions when a minimum interruption diameter of >0.33 mm is chosen. Again, most cortical interruptions detected on HR-pQCT were also detected on µCT (*green arrows*), with some exceptions (*red arrows*). **d** Shows that large cortical interruptions detected on HR-pQCT were not identified as large interruptions on µCT (*red arrows*). **e** Shows that even smaller pores are identified as cortical interruptions when a minimum interruption diameter >0.10 mm was chosen (*green arrows*). (Color figure online)

Figure 3 shows 3D reconstructions of the same MCP joint when examined with different minimum diameters of the interruptions on HR-pQCT and µCT. With both imaging modalities, the cortical bone is very porous and most pores are found on the same location (Fig. 3a). However, in some, thin cortices can be identified on µCT that are not seen on HR-pQCT (Fig. 3a, orange arrows).

When applying the algorithm with a cortical interruption diameter of >0.16 mm on HR-pQCT and µCT, most pores are identified as cortical interruptions and accurately fill the pore in the cortex (Fig. 3b). Most detected interruptions on HR-pQCT are also detected on µCT when the same diameter of >0.16 mm is applied (Fig. 3b, green arrows), with some exceptions (Fig. 3b, red arrows).

When a minimum cortical interruption diameter of >0.33 mm is chosen on HR-pQCT and µCT, only larger pores are identified as cortical interruptions (Fig. 3c). Again, most interruptions detected on HR-pQCT are also detected on µCT (Fig. 3c, green arrows), with some discrepancies (Fig. 3c, red arrows).

Interestingly, the majority of interruptions with a diameter of >0.50 mm on HR-pQCT are not identified as interruptions of the same size on µCT (Fig. 3d, red arrows).

When applying the algorithm with a cortical interruption diameter of >0.10 mm on µCT, even smaller pores are identified as interruptions (Fig. 3e, green arrows). When comparing interruptions with a diameter of >0.16, >0.33 and >0.50 mm on HR-pQCT (Fig. 3b–d) to the interruptions with a diameter of >0.10 mm on µCT (Fig. 3e), we can see that almost all interruptions detected on HR-pQCT are also detected on µCT, although the majority has a smaller diameter (Fig. 3e).

### Reliability at the Joint Level for Different Diameters of the Interruptions

Table 2 shows that the mean number of interruptions detected per joint depends on the diameter cut-off applied and ranges from 3.4 to 53.5 on HR-pQCT and from 1.8 to 45.1 on µCT for interruptions >0.50 and >0.16 mm, respectively. Furthermore, it can be seen that the number of cortical interruptions per joint differs significantly between HR-pQCT and µCT for all diameter cut-offs (Table 2). The interruption surface per joint only differs significantly for interruptions with a diameter of >0.50 mm (Table 2).

**Table 2:** The number of cortical interruptions and interruption surface per joint detected on HR-pQCT and µCT

Minimum diameter of the interruption	Number of interruptions		*p* value	ICC	Interruption surface		*p* value	ICC
	HR-pQCT	µCT			HR-pQCT	µCT		
>0.16 mm	53.5 (33.9)	45.1 (30.4)	0.01	0.91 (0.65 to 0.97)	44.6 (37.8) mm^2^	46.3 (42.6) mm^2^	0.60	0.93 (0.82 to 0.97)
>0.33 mm	9.5 (9.1)	12.8 (11.7)	0.02	0.81 (0.52 to 0.92)	13.5 (14.5) mm^2^	11.7 (12.9) mm^2^	0.75	0.86 (0.67 to 0.94)
>0.50 mm	3.4 (4.1)	1.8 (2.2)	0.02	0.52 (0.11 to 0.78)	6.5 (8.4) mm^2^	1.6 (2.0) mm^2^	<0.01	0.21 (−0.15 to 0.56)

Values are displayed as mean (standard deviation) and for ICC as value (95% confidence interval)

_*ICC* intra-class correlation coefficient_

For the number of interruptions and interruption surface, almost perfect reliability (ICC ≥ 0.81) at the joint level between HR-pQCT and µCT is found for interruptions with a diameter of >0.16 and >0.33 mm. For large interruptions (>0.50 mm), a fair to moderate reliability is found (ICC 0.21–0.52) (Table 2).

### Reliability at the Level of a Single Interruption

#### Fixed µCT Diameter of >0.10 mm

At the level of a single interruption, the PPV_0.10mm_ is 84.9% for interruptions with a diameter of >0.16 mm, 96.7% for interruptions with a diameter of >0.33 mm, and 100% for interruptions with a diameter of >0.50 mm. The sensitivity_0.10mm_ is 62.4% for interruptions with a diameter of >0.16 mm, 12.7% for interruptions with a diameter of >0.33 mm, and 4.7% for interruptions with a diameter >0.50 mm.

#### Same Minimum Diameter

In addition, the PPV_sd_ is substantial for interruptions with a diameter of >0.16 and >0.33 mm (PPV_sd_ = 66.6 and 66.3%, respectively), but fair for interruptions with a diameter of >0.50 mm (PPV_sd_ = 38.5%). The sensitivity_sd_ was substantial for interruptions with a diameter of >0.16 and >0.50 mm (Sensitivity_sd_ = 79.1 and 73.5%, respectively), and moderate for interruptions with a diameter of >0.33 mm (Sensitivity_sd_ = 49.4%).

## Discussion

In this study, we investigated the reliability of cortical interruptions detected with our semi-automated algorithm on HR-pQCT in comparison to µCT as gold standard. We showed that almost all cortical interruptions detected with the algorithm on HR-pQCT were also detected on µCT on the same location. However, the size of an interruption detected on HR-pQCT can be different from that on µCT. In regions of thin bone, the HR-pQCT might not be able to identify these thin structures and can therefore overestimate the interruption diameter (Fig. 1a, orange arrows)[14]. In regions of dense cortical bone, the HR-pQCT tends to underestimate the interruption diameter due to the lower resolution [19, 20]. On the joint level, almost perfect agreement for the number of interruptions and interruption surface was obtained for interruptions with a minimum diameter of >0.16 and >0.33 mm. As expected, the PPV_0.10mm_ of the detection of a single interruption improved with increasing diameter, and was excellent for >0.33 and >0.50 mm (PPV_0.10mm_ ≥ 97%). However, this improvement in PPV_0.10mm_ comes with the expense of a lower sensitivity_0.10mm_, which was low for interruptions with a minimum diameter >0.33 and >0.50 mm (sensitivity_0.10mm_ ≤12.7%).

The number of interruptions with a minimum diameter of >0.16 mm and interruption surface per joint detected in the present study was substantially higher than observed in a previous study by our study team (53.5 and 44.6 mm^2^ vs. 25.0 and 18.9 mm^2^) [15]. This can be explained by the relatively high mean age of the donors in the present study (85.1 years) compared to the subjects investigated in our previous study (48.8 years) [15]. Due to the old age of the donors and preservation in formalin, the cortices become less mineralized [21]. The Tot.BMD of these specimens was about 25% lower than the average in the normal population (248.5 vs. 327 mgHA/cm^3^, respectively) [13]. In addition, the Ct.BMD and Tb.BMD were respectively 31 and 22% lower compared to the normal population (464.0 and 179.7 mgHA/cm^3^ vs. 593 and 262 mgHA/cm^3^, respectively) [13]. These low mineralized and thin cortices are more likely to represent non-bone voxels after binary segmentation than a high mineralized cortex, leading to more cortical interruptions.

Bone density loss is also observed in patients with RA, thus potential overestimation of the number of interruptions and its size might also be observed in patients with RA. However, the Tot.BMD, Ct.BMD and Tb.BMD in patients with RA were 5–7% lower than in the normal population (308, 566 and 593 mgHA/cm^3^ vs. 327, 593 and 243 mgHA/cm^3^, respectively) [13]. Meaning that potential overestimation is possible in patients with RA compared to healthy controls, but it is not likely to have a large effect, because of the small difference in BMD.

A limitation of our algorithm is that it uses a cortical mask with a constant thickness. The chosen value fitted best with the average cortical thickness in the joint. Although this approach is robust, it introduces a systematic error: a cortical interruption is only detected if non-bone voxels connect the inner and outer surface of the mask with a chosen amount of voxels dependent on the number of dilation steps. In regions where the cortex is thin, part of the underlying fatty bone marrow might be included in the mask as non-bone voxels and subsequently become part of a cortical interruption. This might lead to overestimation of the size of an interruption detected. On the other hand, regions of relatively thick cortical bone will lead to underestimation of the interruption size.

The current investigation of the reliability of cortical interruptions detected with our semi-automated algorithm on HR-pQCT compared to µCT as gold standard is a next step in the assessment of small cortical interruptions in finger joints by HR-pQCT. The algorithm can best be used for the detection of interruptions with a minimum diameter of >0.16 or >0.33 mm on HR-pQCT, because an almost perfect agreement on the number and surface of interruptions with µCT was obtained at the joint level, and the locations of interruptions detected on HR-pQCT well matched with µCT. The choice can depend on the scope of the study, if a higher PPV is preferred, one should consider using interruptions >0.33 mm, if a higher sensitivity is preferred, one should consider using interruptions >0.16 mm.

A next step in the validation of the algorithm is the investigation of the reproducibility of the algorithm on patient scan/re-scan (repositioning) data. Additionally, in patients with RA, loss of cortical bone (i.e. a cortical interruption) is often accompanied with loss of trabeculae [1]. Therefore, it would be interesting to extend the algorithm with determination of trabecular bone voids adjacent to the cortical interruption. Finally, the algorithm could be used in clinical studies in order to determine its potential value in monitoring patients with RA, and discriminating patients with RA early in the disease from healthy controls. The average interruption diameter found in previous studies in patients with RA was 2.2 mm [22, 23]. Small interruptions (<0.50 mm) were found in both healthy controls and patients with RA [8], and interruptions >1.9 mm were found highly specific for patients with RA [8]. In early detection of cortical bone loss in patients with RA, this algorithm has the advantage of including the detection of these small cortical interruptions (<0.50 mm) in a highly reliable manner in comparison to visual scoring which showed fair to moderate reliability [12].

In conclusion, this semi-automated algorithm for detection of cortical interruptions with HR-pQCT in finger joints performed best for interruptions with a minimum diameter of >0.16 or >0.33 mm. Almost perfect reliability was obtained at the joint level and the interruptions matched well with those detected on µCT. This algorithm can be useful for evaluation of cortical interruptions in rheumatic diseases.
